# A Microethnographic and Ethnobotanical Approach to Llayta Consumption among Andes Feeding Practices

**DOI:** 10.3390/foods7120202

**Published:** 2018-12-09

**Authors:** Mailing Rivera, Alexandra Galetović, Romina Licuime, Benito Gómez-Silva

**Affiliations:** 1 Departamento de Educación, Facultad de Educación, Universidad de Antofagasta, Antofagasta 124000, Chile; mailing.rivera@uantof.cl (M.R.); rlicuime1505@gmail.com (R.L.); 2 Departamento Biomédico, Facultad Ciencias de la Salud, and Centre for Biotechnology and Bioengineering, CeBiB, Universidad de Antofagasta; Antofagasta 1240000, Chile; alexandra.galetovic@uantof.cl

**Keywords:** Andean microalgae consumption, Atacama, cyanobacteria, Llayta, microethnography, *Nostoc*

## Abstract

Llayta is a dietary supplement that has been used by rural communities in Perú and northern Chile since pre-Columbian days. Llayta is the biomass of colonies of a *Nostoc* cyanobacterium grown in wetlands of the Andean highlands, harvested, sun-dried and sold as an ingredient for human consumption. The biomass has a substantial content of essential amino acids (58% of total amino acids) and polyunsaturated fatty acids (33% total fatty acids). This ancestral practice is being lost and the causes were investigated by an ethnographic approach to register the social representations of Llayta, to document how this Andean feeding practice is perceived and how much the community knows about Llayta. Only 37% of the participants (mostly adults) have had a direct experience with Llayta; other participants (mostly children) did not have any knowledge about it. These social responses reflect anthropological and cultural tensions associated with a lack of knowledge on Andean algae, sites where to find Llayta, where it is commercialized, how it is cooked and on its nutritional benefits. The loss of this ancestral feeding practice, mostly in northern Chile, is probably associated with cultural changes, migration of the rural communities, and very limited access to the available information. We propose that Llayta consumption can be revitalized by developing appropriate educational strategies and investigating potential new food derivatives based on the biomass from the isolated Llayta cyanobacterium.

## 1. Introduction

**Figure 1:**
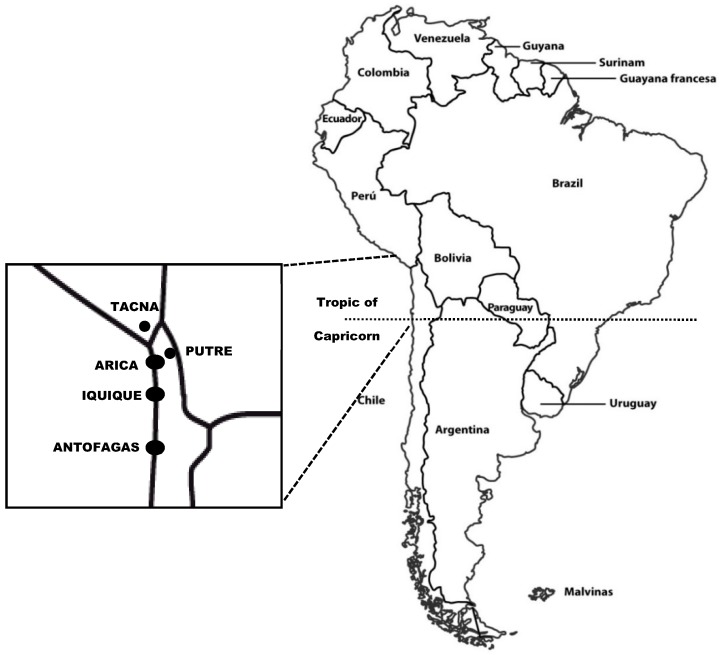
Locations, in southern Peru and northern Chile, where information on Llayta was acquired.

The abundance and diversity of organisms in the Atacama Desert are severely limited by the high levels of desiccation and ultraviolet light [1,2]. In the Andes Mountains highlands, biodiversity is higher and plants have been used for centuries by local communities for feeding, foraging and ethnomedicine [3,4,5,6,7,8]. Microalgae and cyanobacteria are part of the Andes biodiversity but seldom acknowledged. Based on their nutritional and digestive benefits, microalgae and cyanobacteria (i.e., *Chlorella*, *Dunaliella*, *Arthrospira* and *Nostoc*) have been part of the human diet in South America, North America, Asia and Africa. Also, some species are natural resources for a variety of organic molecules with high interest to the biotechnological industry (proteins, amino acids, vitamins, polyunsaturated fatty acids, pigments) [3,4,9,10]. Edible members of the *Nostoc* genus are found in China where *Nostoc flagelliforme* has been consumed as a delicacy for centuries but its collection is prohibited today due to over-exploitation [11,12]. In South America, an indigenous foodstuff harvested in the Andes wetlands, known as Llayta, is the dry biomass of macrocolonies of a cyanobacterium from the genus *Nostoc* (Figure 1). Llayta consumption is a practice that can be traced back to pre-Columbian times and it has been recorded in documents from the 17th century [13,14] and, in a more recent botanical report [15]. 

**Figure 2:**
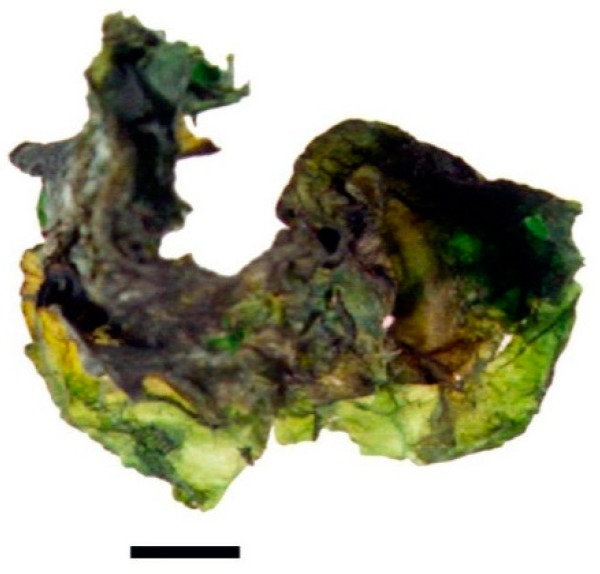
A dry colony of Llayta obtained at a major food market in Arica, Chile. (Bar: 1 cm).

Thus, the genus *Nostoc* has been an old component of the human diet in South America, and it continues to be used today as a food additive in northern Chile (Arica y Parinacota and Tarapacá Regions) and in southern Peru (Tacna City) (Figure 2) [5,6,8,16,17,18]. However, and based on preliminary interviews, this ancient culinary legacy is disappearing and it is already unknown by the urban communities from other areas of the region; for example, at Antofagasta, the major coastal city in northern Chile, nearly 400 km south of Iquique (Figure 2). 

Our report provides the results of a microethnographic study conducted to learn how much people know about Llayta and their perception on this ancestral Andean ingredient, and is meant to be complementary to biochemical studies done on Llayta [4]. This work was based on the following considerations: (i) Llayta consumption is a feeding practice transmitted through generations in the rural Andean world of South America, without reports of adverse effects on human health; (ii) Llayta consumption is an old culinary legacy that is disappearing in regions of South America; (iii) Llayta is a nutritional ingredient containing essential amino acids (58% of total amino acids) and polyunsaturated fatty acids (33% total fatty acids); (iv) the prevalence of undernourishment in South America; (iv) Llayta consumption can be revitalized with appropriate educational strategies and new food derivatives can be developed from the biomass of the isolated cyanobacterium from Llayta [4,9,18,19]. 

We propose that this microethnographic approach will help us to explore explanations for an apparent decrease in Llayta consumption, and to provide arguments and suggestions for the revitalization of this feeding practice.

## 2. Materials and Methods 

### 2.1. The Microethnographic Study 

The microethnographic study on Llayta was designed to learn about the direct or indirect knowledge people have about Llayta by collecting social representations [20] from interviewing participants, including drawings prepared by children. The main expressions about Llayta were ethnographically registered and analyzed in order to explain the social worlds built by persons about their understanding of the surrounding natural, social and cultural environment [21].

### 2.2. Sample for the Microethnographic Study

Observations were carried out during the first half of 2014 in Tacna (Peru) and Putre (Chile) (Figure 2). Tacna is a Peruvian city located at the frontier between Peru and Chile with an active commercial exchange with Arica and Iquique, two coastal cities in Chile. Putre is a rural village at 2500 m above sea level in the Andes Range in northern Chile, close to sites where Llayta is harvested. 

The participants were 19 active members of their community who were informed about the origin and purpose of the study. Their selection was based on their willingness to participate anonymously in the interviews.

The participants were 12 fourth-grade students (7 boys and 5 girls, 9–10 years old) and three middle-aged adults (three males and two females) from Putre, Chile. Participants from Tacna, Peru, were two vendors (one female and one male) working at the main food market of the city. Interviews and observations were conducted at sites normally used by the participants (street, market place, school, and hotel). 

Oral interviews were conducted with all participants in order to learn their direct or indirect knowledge about Llayta (origin, physical description, uses, places where Llayta grows and is sold, its quality as food). In addition, children were also asked to draw representations of Llayta in order to evaluate how close their depictions were from the real subject.

## 3. Results

### 3.1. The Vocable Llayta: Alternative Names and Their Meaning 

Llayta is the Aymara name that refers to colonies of a cyanobacterium that grows in the Andes highlands and is consumed by rural and urban communities in South America (Figure 1 and Figure 2). Alternative names for Llayta can be found in several languages: Spanish, Quechua, Kunza and Mapudungun (Table 1). The variety of names for the vocable Llayta stresses the cultural and anthropological diversity of representations associated with this feeding practice.

### 3.2. How Much People Know about Llayta

All participants were interviewed to assess the type and level of knowledge they have on Llayta. Table 2 provides extracts of the answers given by 10 participants (7 adults and 3 students). Only 8 of the 19 participants had some perception about Llayta; the others (58%) lack any knowledge about it. The extracts in Table 2 corroborate that 7 participants have had personal experience of Llayta, i.e., direct knowledge. Only one teacher expressed indirect knowledge about Llayta since the information was second-hand (Table 2). Compared with adult participants, the oral expression of knowledge used by the fourth-grade students from Putre to refer to Llayta were few or absent. When asked to draw an image of Llayta, 11 out of 12 students were willing to participate and their drawings were far from a correct depiction of the colonies. As an exception, one student emphasized that his mother used to cook Llayta and her drawing was the closest image to it. Another student said: “*no*, *yo no*” (No, I do not (know Llayta)). A third student asked: “¿*Esa es la Llayta*? (Is this Llayta?), referring to a drawing made by another student. Table 2 is a compilation of the representations of Llayta.

### 3.3. Fields of Representations for Llayta

Table 2 shows the extracts from the ethnographic registries. These are the descriptions and references that sustain the field of representation of Llayta for 7 adult participants, which can be organized in the following 3 semantic fields: 

#### 3.3.1. What is it? 

“Es un musgo que se trae de Caquena”; “es un musgo parecido al cochayuyo, es la misma que venden en el agro”. “Ésta es nacional”; “la Llayta es un tipo de alga de mar, de agua dulce y de mar también hay”. 

“It is a moss brought from Caquena”; “it is a moss similar to cochayuyo (seaweed), it is the same that is sold at the market”, “This is national”; “Llayta is a kind of marine alga; from freshwater and also from the sea”. 

#### 3.3.2. Where is it from?

“De súcuro se trae”, “De súcuro de ahí al fondo pues”; “Ahí arriba de Puno”; “ésta es de río”; “en Parinacota hay río de esa, ahí florece”; “la Llayta es de por acá también”; “Hay en las lagunas, en Caqueña en Tacna y aquí arriba Caquena”; “de la altura, de Puno, de Juliaca, bofedales eso está en la altura, en agua dulce en los ríos crece por ahí, bueno acá en la frontera con Perú, tripartito, Visviri, ahí también crece”; “Llayta, arriba hay, arriba”; “Parinacota, ahí si hay Llayta”; “la Llayta es de por acá también”; “en Caquena”; “Eh…Yo no las he visto pero sí me han dicho que ahí en la laguna está la Llayta, pero de verla no”; “en Caquena”. 

“It is brought from Sucuro”; “from Sucuro, back there”; “from Puno, up there”; “this is from a river”; “at Parinacota, there is a river where it blooms”; “Llayta is from here too”; “it is from ponds, at Caquena, in Tacna, and up here in Caquena”; “from highlands, at Puno, Juliaca, wetlands, this is at the highlands, it grows in freshwater rivers, well, here at the border with Peru, Visviri, where it also grows; “Llayta, it is up there, up high”; “Llayta is at Parinacota, for sure”; “Llayta is from here too”; “at Caquena”; “Eh…, I have not seen it but I was told that over there at the pond, there is Llayta, but I have not seen it”; “at Caquena”. 

#### 3.3.3. What is it for? 

“Para picante”; “si picante prepara rico ahí comen”; “los peruanos los comen la Llayta y el cochayuyo”; “Llayta come... el segundo come bonito, así que coce para… es como carne para… se prepara eso”; “como carne, como picante cocino acá”; “la Llayta se usa para el picante”; “la he comido no más, pero no sé qué me ha hecho”; “¡no! y los picantes de guatita”; “ahí le ponen la Llayta…”; “lo traen y lo hacen secar, y seco lo venden en los negocios para cocinarlo se la remoja”; “ah yo sí, porque mi mamá cocina”. 

“For making picante dish”; “yes, picante dish is good, they eat it”; “Peruvian people eat Llayta and cochayuyo”; “Llayta is for consumption…, so you cook it…, it is like meat…; like meat, like picante I cook it here”; “Llayta is used for picante”; “I have eaten it but I do not know how to prepare it”; “it is brought here, it is dried and it is sold dry to restaurants”; “it is soaked before cooking”; “yes I know it, my mother cooks it”.

When asked for places where Llayta can be found, one informant from Tacna, Peru, used the vocable “*chuncuru*” (an Aymara synonym for Llayta) instead of using “*Sucuru*”, the right geographic site to where Llayta can be found. This mistake can be explained by the phonetic similarity between both words. 

## 4. Discussion 

### 4.1. Microethnographic Aspects

The ethnographic goal of this study was to document the anthropological and cultural tensions found in the social representations of the Llayta feeding practice and relate them to the knowledge and value given by the communities to Andean algae.

Ethnographic registries allow the collection of evidence and social representations from people on a particular subject, to discover what people think, believe, and know about their surroundings, and understand how people see it and fit it in their particular interpretations of realities [21,25,26]. The knowledge people may have on a particular natural situation is a good example of where social representations can be collected and interpreted from social and cultural perspectives [26]. Also, descriptions and references from participants are essential in the field of representation for an event of ethnographic interest [22]. 

In the study of the Llayta feeding practice, the ethnographic registry can be supported by anthropological, socio-cultural, and nutritional referents [20]. The first two provide information on the meaning(s) of the term Llayta, the identification of sites where Llayta grows naturally, and where it is commercialized and consumed. 

Our results indicated that nearly 40% of the participants declared they knew Llayta and described it as a moss or an alga, without clarifying whether it grows in fresh water or seawater (Table 2). They also provide names for the sites of origin of Llayta: Parinacota, Putre, Caquena and Visviri in Chile, and Sucuro, Juliaca and Puno in Peru (Table 2, Figure 2). The participants knew that Llayta is used to prepare “*picante*”, a typical dish from rural areas in the Andes; however, they were unaware of its nutritional properties. All fourth-grade students from Putre did not know Llayta (11 out of 12 students; 58% of the participants of this study). 

It is of considerable concern to confirm that a large proportion of the participants, all young people, did not know Llayta. This apparent loss may be explained by considering the impact of new technologies on rural life, cultural changes and migration from rural regions into urban centers, as described for the Aymara people in northern Chile [24,27].

The cultural and anthropological tensions observed in this study stemmed from a lack of knowledge on the following subjects: (a) conceptual limitations to explain what is an Andean alga or a microalga; (b) geographic locations where Llayta can be found and commercialized; (c) weak descriptions to explain how Llayta is cooked; and (d) the benefits obtained from its consumption. Also, this low or absence of knowledge on Llayta can be explained by how people perceive and relate to the world around them and, most stressfully, by a probable extinction of an Andean cultural practice. The massive ignorance about Llayta found in discussions with children in Putre is a clear example of it. 

These anthropological tensions are indication of a paucity of information on Llayta. There is therefore an urgent need to educate people about all the cultural and nutritional knowledge accumulated on Llayta, so that it can be properly valued as a nutritional foodstuff. 

### 4.2. Biochemical Characterization of Llayta 

Llayta consumption is a traditional practice whose future revitalization can be supported by ethnographic information and evidence on the nutritional quality of Llayta [4]. 

The first biochemical evaluation of colonies of *Nostoc* cells from Peruvian wetlands were published by Aldave-Pajares [16,17]. Later, Gómez-Silva et al. [18] registered the proximal composition of Llayta colonies sold and consumed in Chilean territory, and also for the biomass from the isolated Llayta cyanobacterium. Both studies are in agreement on the total protein (30–35% *w/w*) and carbohydrate (50–60% *w/w*) content of the Andean *Nostoc* biomasses. More recently, it was informed that 60% of Llayta amino acids can be classified as indispensable; total lipids accounted for 2% of the biomass dry weight; and 32% of total fatty acids were polyunsaturated fatty acids, Vitamin E content was 4.3 mg% *w/w*, total polyphenols was 64 mg (as equivalent to gallic acid), with an antioxidant activity of 17.4 μmoles (as equivalent to Trolox), and total fiber content was 56% of dry weight [4]. Galetovic et al. [4] inferred that Llayta biomass is a nutritious dietary ingredient. 

One reason for the need for safety assessments of foods and food ingredients based on microalgae and cyanobacteria biomasses to protect public health is the potential presence of cyanobacterial toxins active at low doses (e.g., microcystin and nodularin). In particular, *Arthrospira platensis* (Spirulina) is considered a safe food based on centuries of human consumption [23]. Comparatively, some species of the *Nostoc* genus have toxic members that synthesize microcystine-like cyanotoxin [28]. However, the genome of the colony-forming *Nostoc* strain isolated from the *Llayta* biomass did not show the presence of *mycE*, a key gene in the microcystine biosynthetic pathway, rendering it as a non-toxic *Nostoc* strain [4,28]. In addition, the absence of epidemiological records associated with Llayta consumption diminishes but does not remove the potential presence of toxic secondary metabolites in this cyanobacterial biomass [4].

## 5. Conclusions

Llayta consumption is a feeding practice with a know-how that has been transmitted through generations in the rural Andean world of South America, without untoward effects on human health. Nevertheless, this ancestral feeding legacy is being lost in young generations from northern Chile. New educational and anthropological strategies must be developed if there is interest in promoting the preservation and value of this traditional feeding practice and cultural legacy.

Llayta is a nutritious dietary ingredient for human consumption. This is supported by the absence of adverse epidemiological evidence, but also with interdisciplinary studies that complement ethnographic records with biochemical information. 

Caution on the amount of Llayta consumed daily, based on its arsenic content, must be stressed. However, mass growth of the cyanobacterium isolated from Llayta under controlled conditions would provide arsenic-free biomass for the formulation of new food products. This biotechnological approach would revitalize the use of and add value to this ancestral food ingredient for the benefit of not only Andean communities, but also the whole population of South America and the world.

## Figures and Tables

**Table 1:** The Llayta vocable: alternative names, their ethnic origins and meaning.

Name	Language	Comments	Reference
Cushuro Llullucha Murmuta Crespito Yrurupa		The author did not indicate the language of origin of the words.	[12]
Chuncoro Murmunta	Aymara	“Vna yerua negra de comer, frutilla fe llama Chun-coro,o Murmunta” (Edible black herb called Chuncoro or murmuta)	[3]
Murmunta Chuncuru	Aymara	“(Prunus capulí Cav.) Cerezo. La infusión de las hojas de esta planta se usa como laxante. Sus frutos en Aymara se llaman”. (The leaves infusion is used as a laxative. Their fruit are named in Aymara)	[22]
Cerezo	Aymara	“s. Cerezo. Plumas coloridas (10)” (Colored feathers)	[23]
Quchayuyo Murmunta	Aymara	“Bot. Cerezo. 2. Plumas coloridas, en Bolivia. Bot. Cochayuyo de agua dulce y del mar (p.e. alga comestible). Vte. QUCHAYUYU, MURMUNTA” (Colored feathers in Bolivia. Bot. Macroalga from freshwater and sea water) (edible alga))	[24]
Chungullu	Quechua	“una cianobacteria comestible, se encuentra en riachuelos y lagunitas del bofedal ubicado entre Isluga y Colchane, en las cercanías de la frontera entre Bolivia y la Región de Tarapacá en Chile”. (An edible cyanobacterium, found in small rivers and lakes of wetlands located between Isluga and Colchane, near the border between Bolivia and the Tarapacá Region in Chile)	[7]
Murmunta, Chuncuro	Aymara Quechua	“…, probablemente derivado de su morfología, hábitat y uso. En Aymara: hierba de las ciénagas como granillos negros”. (… derived probably from its morphology, habitat and use. In Aymara: black grain herb from wetlands)	[7]
Luche (lucha)	Mapuche	“…, es un símil de una alga roja, marina y comestible”. (..., is like a marine edible red alga)	[7]
Yullucha	Aymara	“De llullu para referirse a formas vegetales que empiezan a desarrollarse o se pasman”. (From llullu, to refer to plants starting or have stopped their growth).	[8]
Tchuckula Chucula	Kunza	“Nombre atacameño de la cianobacteria comestible Nostoc. Planta acuática que hay en la cordillera”. (Name given by the Atacameños people to the edible cyanobacterium Nostoc. Aquatic plant found in the Andes Range).	[8]
Yoyo	Aymara Quechua	“Cianobacterias del género Nostoc” (cianobacteria from the genus Nostoc).	[9]
Chungulle, chungullo	Quechua	“Cianobacteria acuática de bofedales, procedentes de Chela. Se usa para la comida (caldo con papas chuño). Se indicó que “hay uno que se come, es especial. Se lava y se seca”. (Aquatic cyanobacterium from Chela wetland. It is used as food, in soups with potatoes. It was mentioned that there is an edible one which is special. It is washed and dried).	[9]
Luche	Mapuche	“Símil de un alga roja comestible para designar a un alga verde-azulada de agua dulce, también comestible (Nostoc)”. (Similar to an edible red alga, it is (Used to indicate an edible, freshwater blue-green alga, (Nostoc)).	[9]
Yoyo	Aymara	“Nombre de la cianobacteria Nostoc en Ollagüe, posiblemente aludiendo a su carácter comestible. Planta acuática comestible”. (Name given in Ollagüe to the cyanobacterium Nostoc, possibly due to its edibility).	[9]

**Table 2:** Transcripts of interviews conducted to adult and students participants about their knowledge on Llayta.

Informant	Type of Knowledge about Llayta
**DIRECT KNOWLEDGE:**	
Saleswoman at the food market, Tacna, Peru.	“Ahí huapé súcuros; de Súcuro se trae; de súcuro de ahí al fondo pues; ahí arriba de Puno. Otros caballeros traen y ahí compramos; para picante. Si, picante prepara rico ahí comen”. (There, huapé súcuros; it is brought from Sucuro; down there; there, above Puno. Other people bring it and we buy it to make “picante”—A local dish; yes, a tasty picante is prepared up there).
Salesman at the food market, Tacna, Peru.	“Esta es nacional; ésta la traen de Camaná. Esta es de río muestra -y muestra Llayta-, es más rica y esa es de mar -muestra cochayuyo; a tres soles. Esto jefe chángalo, muélelo, jugo”. (This is Peruvian; it is brought from Camaná. This from a river, it is better and this one is from the sea; it is worth three Peruvian new sols. Cut it and grind it for juice, boss).
Professional cook, Chile.	“Para el consumo lo comemos eh; la Llayta es un tipo de alga de mar, de agua dulce y de mar también hay. Lo traen y lo hacen secar, y seco lo venden en los negocios; para cocinarlo se la remoja. Los peruanos los comen la Llayta y el cochayuyo. De la altura, de Puno, de Juliaca, bofedales eso está en la altura, en agua dulce en los ríos crece por ahí, bueno acá en la frontera con Perú, tripartito, Visviri, ahí también crece”. (We eat it for consumption, eh!; Llayta is a kind of marine alga; it is from freshwater and also marine. They bring it, dry it and sell it dried; they soak it before cooking. Peruvians eat Llayta and cochayuyo. From the highlands, from Puno, from Juliaca, from wetlands, which are in the highlands, in freshwater rivers, it grows around; right here, at the three parties’ border, it also grows at Visviri).
Middle age woman, Putre, Chile.	“Llayta, arriba hay, arriba; Parinacota, ahí si hay Llayta; Llayta come..., el segundo come bonito, así que coce para…, es como carne para…, se prepara eso como carne, como picante cocino acá. Ahí no se pa’ qué sea, en Parinacota hay río de esa, ahí florece”. (Llayta is from up there; Llayta is at Parinacota; Llayta is eaten…, nice as a second dish…, it is cooked…, it is like meat…, it is prepared as meat…, I cook it here as picante. I do not know what it is used for over there; in Parinacota there is a river where it grows).
Director, school at Putre, Chile.	“La Llayta es de por acá también. La Llayta se usa para el picante. La he comido no más, pero no sé qué me ha hecho; acá hacen mucho picante de pata con guata y Llayta. Hay en las lagunas, en Caqueña, en Tacna y aquí arriba Caquena”. (Llayta is from here too. Llayta is used in picante. I have only eaten it; I do not know the effects on me; people right here prepares a lot of picante with meat and Llayta. There are some ponds, in Casqueña, in Tacna and up here in Caquena).
Teacher 1, at school in Putre, Chile.	“¡no! y los picantes de guatita, ahí le ponen la Llayta… es un musgo que se trae de Caquena; es un musgo parecido al cochayuyo, es la misma que venden en el agro”. (No! Llayta is added at the picante dishes…, it is a moss brought from Caquena; it is a moss similar to cochayuyo marine macroalga, it is the same one that is sold at the food market).
Student 1, at school in Putre, Chile.	“Ah! yo sí, porque mi mamá cocina. En Caquena”. (Mm! I do, since my mother cooks it. At Caquena…).
**INDIRECT KNOWLEDGE:**	
Teacher 2 at school in Putre, Chile.	“Eh…Yo no las he visto pero sí me han dicho que ahí en la laguna está la Llayta” pero de verla no. En Caqueña en Tacna y aquí arriba Caquena”. (Eh…, I have not seen it, but I have been told that Llayta is at the pond, but I have not seen it. At Caquena, in Tacna and up here in Caquena).
**WITHOUT KNOWLEDGE:**	
Student 2, at school in Putre, Chile.	“No, yo no”. (No, I do not…know Llayta).
Student 3, at school in Putre, Chile.	“¿esa es la Llayta?” (Is that Llayta?)
